# viRome: an R package for the visualization and analysis of viral small RNA sequence datasets

**DOI:** 10.1093/bioinformatics/btt297

**Published:** 2013-05-24

**Authors:** Mick Watson, Esther Schnettler, Alain Kohl

**Affiliations:** ^1^ARK-Genomics, The Roslin Institute and Royal (Dick) School of Veterinary Studies, University of Edinburgh, Easter Bush, Midlothian EH25 9RG and ^2^MRC-University of Glasgow Centre for Virus Research, 8 Church Street, Glasgow G11 5JR, UK

## Abstract

**Summary:** RNA interference (RNAi) is known to play an important part in defence against viruses in a range of species. Second-generation sequencing technologies allow us to assay these systems and the small RNAs that play a key role with unprecedented depth. However, scientists need access to tools that can condense, analyse and display the resulting data. Here, we present viRome, a package for R that takes aligned sequence data and produces a range of essential plots and reports.

**Availability and implementation:** viRome is released under the BSD license as a package for R available for both Windows and Linux http://virome.sf.net. Additional information and a tutorial is available on the ARK-Genomics website: http://www.ark-genomics.org/bioinformatics/virome.

**Contact:** mick.watson@roslin.ed.ac.uk

## 1 INTRODUCTION

RNA interference (RNAi) is mediated by small RNAs, such as micoRNAs (miRNAs) of 21–22 nt (Lagos-Quintana *et al.*, 2001), small interfering RNAs (siRNAs) of 21–22 nt (Bernstein *et al.*, 2001; Zamore *et al.*, 2000) and PIWI-interacting RNAs (piRNAs) of 24–30 nt (Aravin *et al.*, 2003; Brennecke *et al.*, 2007), and these molecules regulate many biological processes. These pathways are also a major part of the antiviral response in both insects and plants, including a variety of important mosquito-borne diseases of humans and animals, such as West Nile Virus, Dengue Virus and Chikungunya Virus. In arthropods, these are characterized by the production of 21–22 nt virus-derived small interfering RNAs (viRNAs) or 24–30 nt viral piRNA-like molecules (Blair, 2011; Donald *et al.*, 2012; Myles *et al.*, 2009).

Second-generation sequencing allows scientists to assay these systems in unprecedented depth, and short reads capture both the 21–22 nt siRNAs and the 24–30 nt piRNAs. However, there is a need for scientists to be able to summarize, analyse and visualize the results of such experiments. Here, we present viRome, a package for R, which takes aligned sequencing data in the BAM format (Li *et al.*, 2009) and produces a variety of plots and reports that are essential to the analysis of data from viral siRNA datasets.

Software packages to analyse viral siRNA data exist. Paparrazi (Vodovar *et al.*, 2011) is designed to reconstruct viral genomes from siRNA data and produces some similar plots to viRome. Alternatively, Visitor (Antoniewski, 2011), an informatic pipeline for analysing short-read viRNA data, also produces several similar plots. However, both are implemented in Perl and are limited to the Linux/Unix operating system; they include alignment as part of the analysis; therefore, using an alternative aligner would require programming skills; finally, the plots are generated in batch mode; hence, there is no interaction between the user and the software.

As a package for R, viRome improves on these software packages in several ways, including (i) viRome allows interaction between the user and the software during report and graph generation, (ii) viRome is available on any operating system that supports R and has been tested on Microsoft Windows and several Linux distributions, (iii) viRome separates visualization from alignment; therefore, the user is free to use any alignment software they wish and (iv) as an R package, viRome integrates seamlessly with other R packages from the Bioconductor project (Gentleman *et al.*, 2004).

## 2 ANALYSIS AND VISUALIZATION

**Fig. 1.**
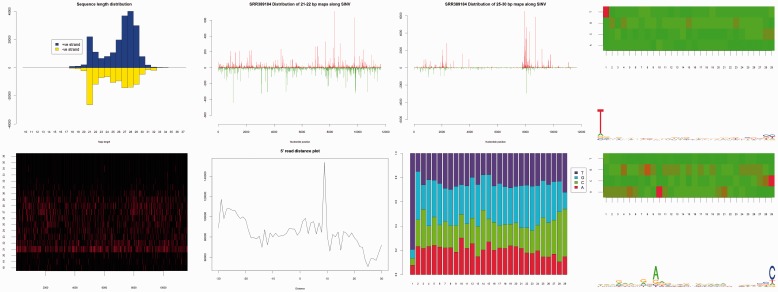
Clockwise from top-left: a plot of read-length distribution; genomic location of 21–22 nt reads; genomic location of 25–29 nt reads; heatmap and sequence logo showing T_1_ bias; heatmap and sequence logo showing A_10_ bias; barplot showing T_1_ bias; 5′ read distance plot for 25–29 nt reads showing enrichment of 10 nt overlap; and a heatmap showing the genomic location of 18–36 bp reads (counts per position: black is low, red is high)

As input, viRome takes aligned sequence data in the BAM format. Many tools exist for alignment (Fonseca *et al.*, 2012) and provided they support the SAM/BAM format, viRome is capable of working with their output. Many of the functions within viRome attempt to summarize millions of data points into tables and plots that allow biological interpretation. One of the benefits of viRome is that most functions return the summarized data, as well as creating a plot. This allows users to create their own plots if they wish. Figure 1 shows a selection of plots produced by viRome.

**Global analyses:** One of the first requirements is to plot a histogram of the lengths of mapped reads—a peak at 21–22 nt implying an siRNA response, and a high frequency of 24–30 nt with a peak at 28 a piRNA response. In viRome, this can be created using the *barplot.bam* function. Users may also create a report using the *sequence.report* function. This produces a data.frame in R that summarizes and counts the sequences aligned to each base in a given reference sequence. Users can see the exact sequence, its length, the location and strand of the alignment plus a count of how many times that sequence occurs. As a data.frame, this can be easily exported to Excel or other spreadsheet software.

**Location-based analyses:** Although many viruses are targeted by the siRNA pathway throughout the genome, others are targeted only in limited regions (Sabin *et al.*, 2013). A heatmap representing the occurrence of all mapped read lengths across all genomic locations can be produced using the *size.position.heatmap* function, and barplots showing counts for each genomic location for each read length generated using the *stacked.barplot* function.

**Read-based analyses:** Read-based analyses allow users to focus on patterns in particular subsets of reads. Single barplots showing the location, strand and count of reads mapping throughout the genome can be visualized using the *position.barplot* function. The base composition of subsets of reads can be calculated with the *make.pwm* function. Sequence signatures of the piRNA pathway include a strong U_1_ bias in primary, antisense piRNAs and following ‘ping-pong’ cycle amplification involving AGO3 and Aub, a strong A_10_ bias in secondary sense piRNAs in *Drosophila* (Brennecke *et al.*, 2007). Similar motifs have been found in piRNAs and viral piRNA-like molecules in mosquitoes or derived cell lines (Morazzani *et al.*, 2012; Schnettler *et al.*, 2013; Vodovar *et al.*, 2012). The output of *make.pwm* can be plotted as a heatmap using the *pwm.heatmap* function, or used with external packages such as seqLogo and motifStack to produce sequence logos. Finally, the 5′-ends of complementary piRNAs are most frequently separated by 10 nt (Brennecke *et al.*, 2007; Vodovar *et al.*, 2012) because of the earlier described ‘ping-pong’ amplification. The distance between 5′-ends of piRNAs mapping to opposite strands can be summarized and visualized using the *read.dist.plot* function.

## 3 CONCLUSIONS

Deep sequencing experiments have revealed a variety of interesting and unique signatures of the miRNA, siRNA and piRNA pathways, and there is a need for software that allows scientists to process such data. We have developed viRome, a package for R that allows the interactive generation of a range of informative plots and reports. As an R package, viRome is available on a range of operating systems. viRome is released under an open-source license and can be downloaded from http://virome.sf.net, where a tutorial is also available.

*Funding*: UK Biotechnology and Biological Sciences Research Council (BBSRC) (BB/J004243/1; BB/J004235/1) (to M.W.); UK Medical Research Council (MRC) (to A.K. and E.S.); The Netherlands Organisation for Scientific Research NWO (Rubicon Fellowship number: 825.10.021) (to E.S.).

*Conflict of Interest*: none declared.

## References

[btt297-B1] Antoniewski C (2011). Visitor, an informatic pipeline for analysis of viral siRNA sequencing datasets. Methods Mol. Biol..

[btt297-B2] Aravin AA (2003). The small RNA profile during Drosophila melanogaster development. Dev. Cell.

[btt297-B3] Bernstein E (2001). Role for a bidentate ribonuclease in the initiation step of RNA interference. Nature.

[btt297-B4] Blair CD (2011). Mosquito RNAi is the major innate immune pathway controlling arbovirus infection and transmission. Future Microbiol..

[btt297-B5] Brennecke J (2007). Discrete small RNA-generating loci as master regulators of transposon activity in *Drosophila*. Cell.

[btt297-B6] Donald CL (2012). New insights into control of arbovirus replication and spread by insect RNA interference pathways. Insects.

[btt297-B7] Fonseca NA (2012). Tools for mapping high-throughput sequencing data. Bioinformatics.

[btt297-B8] Gentleman RC (2004). Bioconductor: open software development for computational biology and bioinformatics. Genome Biol..

[btt297-B9] Lagos-Quintana M (2001). Identification of novel genes coding for small expressed RNAs. Science.

[btt297-B10] Li H (2009). The sequence alignment/map format and SAMtools. Bioinformatics.

[btt297-B11] Morazzani EM (2012). Production of virus-derived ping-pong-dependent piRNA-like small RNAs in the mosquito soma. PLoS Pathog..

[btt297-B12] Myles KM (2009). Origins of alphavirus-derived small RNAs in mosquitoes. RNA Biol..

[btt297-B13] Sabin LR (2013). Dicer-2 processes diverse viral RNA species. PloS One.

[btt297-B14] Schnettler E (2013). RNA interference targets arbovirus replication in culicoides cells. J. Virol..

[btt297-B15] Vodovar N (2011). In silico reconstruction of viral genomes from small RNAs improves virus-derived small interfering RNA profiling. J. Virol..

[btt297-B16] Vodovar N (2012). Arbovirus-derived piRNAs exhibit a ping-pong signature in mosquito cells. PloS One.

[btt297-B17] Zamore PD (2000). RNAi: double-stranded RNA directs the ATP-dependent cleavage of mRNA at 21 to 23 nucleotide intervals. Cell.

